# Supplementary material for: Differences in the acceptance of wife abuse among ethnic minority Garo and Santal and mainstream Bengali communities in rural Bangladesh
Source: PLoS One. 2020 Jul 28;15(7):e0236733. doi: 10.1371/journal.pone.0236733 (PMC7386579; doi:10.1371/journal.pone.0236733)
Supplement: S1 File — (DOCX) [file pone.0236733.s001.docx]

GET

FILE='F:\Lineas\Paper 2\Baseline Data May 2019_1 (04-7-19 Final).sav'.

DATASET NAME DataSet1 WINDOW=FRONT.

* Generalized Linear Models.

GENLIN AcPhy BY Ethnicity Sex Age.c Edu.c Occu Familyinc.c Structure Residence QCL_Moblty EmpQCL_1

(ORDER=ASCENDING)

/MODEL Ethnicity Sex Age.c Edu.c Occu Familyinc.c Structure Residence QCL_Moblty EmpQCL_1

INTERCEPT=YES

DISTRIBUTION=POISSON LINK=LOG

/CRITERIA METHOD=FISHER(1) SCALE=1 COVB=ROBUST MAXITERATIONS=100 MAXSTEPHALVING=5

PCONVERGE=1E-006(ABSOLUTE) SINGULAR=1E-012 ANALYSISTYPE=3(WALD) CILEVEL=95 CITYPE=WALD

LIKELIHOOD=FULL

/EMMEANS SCALE=ORIGINAL

/EMMEANS TABLES=Ethnicity SCALE=ORIGINAL

/MISSING CLASSMISSING=EXCLUDE

/PRINT CPS DESCRIPTIVES MODELINFO FIT SUMMARY SOLUTION (EXPONENTIATED).

**Generalized Linear Models**

| **Notes** | | |
| --- | --- | --- |
| Output Created | | 21-MAY-2020 18:10:18 |
| Comments | |  |
| Input | Data | F:\Lineas\Paper 2\Baseline Data May 2019_1 (04-7-19 Final).sav |
|  | Active Dataset | DataSet1 |
|  | Filter | <none> |
|  | Weight | <none> |
|  | Split File | <none> |
|  | N of Rows in Working Data File | 1929 |
| Missing Value Handling | Definition of Missing | User-defined missing values for factor, subject and within-subject variables are treated as missing. |
|  | Cases Used | Statistics are based on cases with valid data for all variables in the model. |
| Weight Handling | | not applicable |
| Syntax | | GENLIN AcPhy BY Ethnicity Sex Age.c Edu.c Occu Familyinc.c Structure Residence QCL_Moblty EmpQCL_1  (ORDER=ASCENDING)  /MODEL Ethnicity Sex Age.c Edu.c Occu Familyinc.c Structure Residence QCL_Moblty EmpQCL_1  INTERCEPT=YES  DISTRIBUTION=POISSON LINK=LOG  /CRITERIA METHOD=FISHER(1) SCALE=1 COVB=ROBUST MAXITERATIONS=100 MAXSTEPHALVING=5  PCONVERGE=1E-006(ABSOLUTE) SINGULAR=1E-012 ANALYSISTYPE=3(WALD) CILEVEL=95 CITYPE=WALD  LIKELIHOOD=FULL  /EMMEANS SCALE=ORIGINAL  /EMMEANS TABLES=Ethnicity SCALE=ORIGINAL  /MISSING CLASSMISSING=EXCLUDE  /PRINT CPS DESCRIPTIVES MODELINFO FIT SUMMARY SOLUTION (EXPONENTIATED). |
| Resources | Processor Time | 00:00:00.34 |
|  | Elapsed Time | 00:00:00.34 |

[DataSet1] F:\Lineas\Paper 2\Baseline Data May 2019_1 (04-7-19 Final).sav

| **Model Information** | |
| --- | --- |
| Dependent Variable | Acceptance of Physical Abuse |
| Probability Distribution | Poisson |
| Link Function | Log |

| **Case Processing Summary** | | |
| --- | --- | --- |
|  | N | Percent |
| Included | 1929 | 100.0% |
| Excluded | 0 | 0.0% |
| Total | 1929 | 100.0% |

| **Categorical Variable Information** | | | | |
| --- | --- | --- | --- | --- |
|  | | | N | Percent |
| Factor | Ethnicity | Garo | 640 | 33.2% |
|  |  | Santal | 640 | 33.2% |
|  |  | Bengali | 649 | 33.6% |
|  |  | Total | 1929 | 100.0% |
|  | Sex | Male | 960 | 49.8% |
|  |  | Female | 969 | 50.2% |
|  |  | Total | 1929 | 100.0% |
|  | Age in years | 16-25 | 301 | 15.6% |
|  |  | 26-35 | 746 | 38.7% |
|  |  | 36-45 | 711 | 36.9% |
|  |  | 46-60 | 171 | 8.9% |
|  |  | Total | 1929 | 100.0% |
|  | Schooling | Upper | 385 | 20.0% |
|  |  | Secondary | 575 | 29.8% |
|  |  | Primary | 820 | 42.5% |
|  |  | None | 149 | 7.7% |
|  |  | Total | 1929 | 100.0% |
|  | Occupation | Unemployed | 636 | 33.0% |
|  |  | Agric farming | 329 | 17.1% |
|  |  | Day laborers | 797 | 41.3% |
|  |  | Job and others | 167 | 8.7% |
|  |  | Total | 1929 | 100.0% |
|  | Family Income | 15000/above | 478 | 24.8% |
|  |  | 9000-14999 | 852 | 44.2% |
|  |  | Bellow 9000 | 599 | 31.1% |
|  |  | Total | 1929 | 100.0% |
|  | Family structure | Nuclear | 1411 | 73.1% |
|  |  | Joint | 518 | 26.9% |
|  |  | Total | 1929 | 100.0% |
|  | Family residence | Independent | 474 | 24.6% |
|  |  | Matrilocal | 382 | 19.8% |
|  |  | Patrilocal | 1073 | 55.6% |
|  |  | Total | 1929 | 100.0% |
|  | Female Mobility Category | High | 570 | 29.5% |
|  |  | Moderate | 776 | 40.2% |
|  |  | Low | 583 | 30.2% |
|  |  | Total | 1929 | 100.0% |
|  | Female Empowerment | Good | 274 | 14.2% |
|  |  | Fair | 940 | 48.7% |
|  |  | Poor | 715 | 37.1% |
|  |  | Total | 1929 | 100.0% |

| **Continuous Variable Information** | | | | | | |
| --- | --- | --- | --- | --- | --- | --- |
|  | | N | Minimum | Maximum | Mean | Std. Deviation |
| Dependent Variable | Acceptance of Physical Abuse | 1929 | .00 | 10.00 | .7558 | 1.42043 |

| **Goodness of Fit^a^** | | | |
| --- | --- | --- | --- |
|  | Value | df | Value/df |
| Deviance | 2961.526 | 1907 | 1.553 |
| Scaled Deviance | 2961.526 | 1907 |  |
| Pearson Chi-Square | 4283.208 | 1907 | 2.246 |
| Scaled Pearson Chi-Square | 4283.208 | 1907 |  |
| Log Likelihood^b^ | -2313.409 |  |  |
| Akaike's Information Criterion (AIC) | 4670.817 |  |  |
| Finite Sample Corrected AIC (AICC) | 4671.348 |  |  |
| Bayesian Information Criterion (BIC) | 4793.242 |  |  |
| Consistent AIC (CAIC) | 4815.242 |  |  |
| Dependent Variable: Acceptance of Physical Abuse  Model: (Intercept), Ethnicity, Sex, Age.c, Edu.c, Occu, Familyinc.c, Structure, Residence, QCL_Moblty, EmpQCL_1^a^ | | | |
| a. Information criteria are in smaller-is-better form. | | | |
| b. The full log likelihood function is displayed and used in computing information criteria. | | | |

| **Omnibus Test^a^** | | |
| --- | --- | --- |
| Likelihood Ratio Chi-Square | df | Sig. |
| 818.948 | 21 | .000 |
| Dependent Variable: Acceptance of Physical Abuse  Model: (Intercept), Ethnicity, Sex, Age.c, Edu.c, Occu, Familyinc.c, Structure, Residence, QCL_Moblty, EmpQCL_1^a^ | | |
| a. Compares the fitted model against the intercept-only model. | | |

| **Tests of Model Effects** | | | |
| --- | --- | --- | --- |
| Source | Type III | | |
|  | Wald Chi-Square | df | Sig. |
| (Intercept) | 37.071 | 1 | .000 |
| Ethnicity | 28.114 | 2 | .000 |
| Sex | 121.606 | 1 | .000 |
| Age.c | 13.739 | 3 | .003 |
| Edu.c | 18.062 | 3 | .000 |
| Occu | 7.021 | 3 | .071 |
| Familyinc.c | 2.307 | 2 | .315 |
| Structure | .492 | 1 | .483 |
| Residence | 1.454 | 2 | .483 |
| QCL_Moblty | 7.213 | 2 | .027 |
| EmpQCL_1 | .687 | 2 | .709 |
| Dependent Variable: Acceptance of Physical Abuse  Model: (Intercept), Ethnicity, Sex, Age.c, Edu.c, Occu, Familyinc.c, Structure, Residence, QCL_Moblty, EmpQCL_1 | | | |

| **Parameter Estimates** | | | | | | | | | | |
| --- | --- | --- | --- | --- | --- | --- | --- | --- | --- | --- |
| Parameter | B | Std. Error | 95% Wald Confidence Interval | | Hypothesis Test | | | Exp(B) | 95% Wald Confidence Interval for Exp(B) | |
|  |  |  | Lower | Upper | Wald Chi-Square | df | Sig. |  | Lower | Upper |
| (Intercept) | 1.633 | .3009 | 1.043 | 2.223 | 29.459 | 1 | .000 | 5.119 | 2.839 | 9.232 |
| [Ethnicity=1.00] | -.822 | .1603 | -1.136 | -.508 | 26.283 | 1 | .000 | .440 | .321 | .602 |
| [Ethnicity=2.00] | -.400 | .1356 | -.666 | -.135 | 8.716 | 1 | .003 | .670 | .514 | .874 |
| [Ethnicity=3.00] | 0^a^ | . | . | . | . | . | . | 1 | . | . |
| [Sex=1.00] | -1.664 | .1509 | -1.960 | -1.369 | 121.606 | 1 | .000 | .189 | .141 | .254 |
| [Sex=2.00] | 0^a^ | . | . | . | . | . | . | 1 | . | . |
| [Age.c=1.00] | -.659 | .2099 | -1.071 | -.248 | 9.869 | 1 | .002 | .517 | .343 | .780 |
| [Age.c=2.00] | -.693 | .1881 | -1.061 | -.324 | 13.560 | 1 | .000 | .500 | .346 | .723 |
| [Age.c=3.00] | -.533 | .1806 | -.887 | -.179 | 8.694 | 1 | .003 | .587 | .412 | .836 |
| [Age.c=4.00] | 0^a^ | . | . | . | . | . | . | 1 | . | . |
| [Edu.c=1.00] | -.664 | .1625 | -.983 | -.346 | 16.714 | 1 | .000 | .515 | .374 | .708 |
| [Edu.c=2.00] | -.281 | .1423 | -.560 | -.003 | 3.914 | 1 | .048 | .755 | .571 | .997 |
| [Edu.c=3.00] | -.284 | .1346 | -.548 | -.020 | 4.442 | 1 | .035 | .753 | .578 | .980 |
| [Edu.c=4.00] | 0^a^ | . | . | . | . | . | . | 1 | . | . |
| [Occu=1.00] | .049 | .1914 | -.326 | .424 | .066 | 1 | .798 | 1.050 | .722 | 1.528 |
| [Occu=2.00] | .365 | .2099 | -.047 | .776 | 3.017 | 1 | .082 | 1.440 | .954 | 2.172 |
| [Occu=3.00] | .286 | .1927 | -.091 | .664 | 2.208 | 1 | .137 | 1.332 | .913 | 1.943 |
| [Occu=4.00] | 0^a^ | . | . | . | . | . | . | 1 | . | . |
| [Familyinc.c=1.00] | -.171 | .1129 | -.393 | .050 | 2.307 | 1 | .129 | .842 | .675 | 1.051 |
| [Familyinc.c=2.00] | -.062 | .0932 | -.245 | .120 | .446 | 1 | .504 | .940 | .783 | 1.128 |
| [Familyinc.c=3.00] | 0^a^ | . | . | . | . | . | . | 1 | . | . |
| [Structure=1.00] | -.067 | .0960 | -.255 | .121 | .492 | 1 | .483 | .935 | .775 | 1.128 |
| [Structure=2.00] | 0^a^ | . | . | . | . | . | . | 1 | . | . |
| [Residence=1.00] | .062 | .0944 | -.123 | .247 | .425 | 1 | .514 | 1.063 | .884 | 1.280 |
| [Residence=2.00] | .183 | .1579 | -.126 | .492 | 1.345 | 1 | .246 | 1.201 | .881 | 1.636 |
| [Residence=3.00] | 0^a^ | . | . | . | . | . | . | 1 | . | . |
| [QCL_Moblty=1] | -.257 | .1419 | -.535 | .021 | 3.289 | 1 | .070 | .773 | .585 | 1.021 |
| [QCL_Moblty=2] | -.275 | .1045 | -.479 | -.070 | 6.904 | 1 | .009 | .760 | .619 | .933 |
| [QCL_Moblty=3] | 0^a^ | . | . | . | . | . | . | 1 | . | . |
| [EmpQCL_1=1] | -.013 | .1571 | -.321 | .295 | .007 | 1 | .934 | .987 | .725 | 1.343 |
| [EmpQCL_1=2] | -.073 | .0934 | -.256 | .110 | .608 | 1 | .435 | .930 | .774 | 1.117 |
| [EmpQCL_1=3] | 0^a^ | . | . | . | . | . | . | 1 | . | . |
| (Scale) | 1^b^ |  |  |  |  |  |  |  |  |  |
| Dependent Variable: Acceptance of Physical Abuse  Model: (Intercept), Ethnicity, Sex, Age.c, Edu.c, Occu, Familyinc.c, Structure, Residence, QCL_Moblty, EmpQCL_1 | | | | | | | | | | |
| a. Set to zero because this parameter is redundant. | | | | | | | | | | |
| b. Fixed at the displayed value. | | | | | | | | | | |

**Estimated Marginal Means 1: Grand Mean**

| **Estimates** | | | |
| --- | --- | --- | --- |
| Mean | Std. Error | 95% Wald Confidence Interval | |
|  |  | Lower | Upper |
| .6401 | .04690 | .5545 | .7390 |

**Estimated Marginal Means 2: Ethnicity**

| **Estimates** | | | | |
| --- | --- | --- | --- | --- |
| Ethnicity | Mean | Std. Error | 95% Wald Confidence Interval | |
|  |  |  | Lower | Upper |
| Garo | .4229 | .04605 | .3416 | .5235 |
| Santal | .6446 | .08198 | .5024 | .8271 |
| Bengali | .9620 | .10861 | .7711 | 1.2003 |

* Generalized Linear Models.

GENLIN AcPcy BY Ethnicity Sex Age.c Edu.c Occu Familyinc.c Structure Residence QCL_Moblty EmpQCL_1

(ORDER=ASCENDING)

/MODEL Ethnicity Sex Age.c Edu.c Occu Familyinc.c Structure Residence QCL_Moblty EmpQCL_1

INTERCEPT=YES

DISTRIBUTION=POISSON LINK=LOG

/CRITERIA METHOD=FISHER(1) SCALE=1 COVB=ROBUST MAXITERATIONS=100 MAXSTEPHALVING=5

PCONVERGE=1E-006(ABSOLUTE) SINGULAR=1E-012 ANALYSISTYPE=3(WALD) CILEVEL=95 CITYPE=WALD

LIKELIHOOD=FULL

/EMMEANS SCALE=ORIGINAL

/EMMEANS TABLES=Ethnicity SCALE=ORIGINAL

/MISSING CLASSMISSING=EXCLUDE

/PRINT CPS DESCRIPTIVES MODELINFO FIT SUMMARY SOLUTION (EXPONENTIATED).

**Generalized Linear Models**

| **Notes** | | |
| --- | --- | --- |
| Output Created | | 21-MAY-2020 18:11:06 |
| Comments | |  |
| Input | Data | F:\Lineas\Paper 2\Baseline Data May 2019_1 (04-7-19 Final).sav |
|  | Active Dataset | DataSet1 |
|  | Filter | <none> |
|  | Weight | <none> |
|  | Split File | <none> |
|  | N of Rows in Working Data File | 1929 |
| Missing Value Handling | Definition of Missing | User-defined missing values for factor, subject and within-subject variables are treated as missing. |
|  | Cases Used | Statistics are based on cases with valid data for all variables in the model. |
| Weight Handling | | not applicable |
| Syntax | | GENLIN AcPcy BY Ethnicity Sex Age.c Edu.c Occu Familyinc.c Structure Residence QCL_Moblty EmpQCL_1  (ORDER=ASCENDING)  /MODEL Ethnicity Sex Age.c Edu.c Occu Familyinc.c Structure Residence QCL_Moblty EmpQCL_1  INTERCEPT=YES  DISTRIBUTION=POISSON LINK=LOG  /CRITERIA METHOD=FISHER(1) SCALE=1 COVB=ROBUST MAXITERATIONS=100 MAXSTEPHALVING=5  PCONVERGE=1E-006(ABSOLUTE) SINGULAR=1E-012 ANALYSISTYPE=3(WALD) CILEVEL=95 CITYPE=WALD  LIKELIHOOD=FULL  /EMMEANS SCALE=ORIGINAL  /EMMEANS TABLES=Ethnicity SCALE=ORIGINAL  /MISSING CLASSMISSING=EXCLUDE  /PRINT CPS DESCRIPTIVES MODELINFO FIT SUMMARY SOLUTION (EXPONENTIATED). |
| Resources | Processor Time | 00:00:00.25 |
|  | Elapsed Time | 00:00:00.33 |

| **Model Information** | |
| --- | --- |
| Dependent Variable | Acceptance of Emotional Abuse |
| Probability Distribution | Poisson |
| Link Function | Log |

| **Case Processing Summary** | | |
| --- | --- | --- |
|  | N | Percent |
| Included | 1929 | 100.0% |
| Excluded | 0 | 0.0% |
| Total | 1929 | 100.0% |

| **Categorical Variable Information** | | | | |
| --- | --- | --- | --- | --- |
|  | | | N | Percent |
| Factor | Ethnicity | Garo | 640 | 33.2% |
|  |  | Santal | 640 | 33.2% |
|  |  | Bengali | 649 | 33.6% |
|  |  | Total | 1929 | 100.0% |
|  | Sex | Male | 960 | 49.8% |
|  |  | Female | 969 | 50.2% |
|  |  | Total | 1929 | 100.0% |
|  | Age in years | 16-25 | 301 | 15.6% |
|  |  | 26-35 | 746 | 38.7% |
|  |  | 36-45 | 711 | 36.9% |
|  |  | 46-60 | 171 | 8.9% |
|  |  | Total | 1929 | 100.0% |
|  | Schooling | Upper | 385 | 20.0% |
|  |  | Secondary | 575 | 29.8% |
|  |  | Primary | 820 | 42.5% |
|  |  | None | 149 | 7.7% |
|  |  | Total | 1929 | 100.0% |
|  | Occupation | Unemployed | 636 | 33.0% |
|  |  | Agric farming | 329 | 17.1% |
|  |  | Day laborers | 797 | 41.3% |
|  |  | Job and others | 167 | 8.7% |
|  |  | Total | 1929 | 100.0% |
|  | Family Income | 15000/above | 478 | 24.8% |
|  |  | 9000-14999 | 852 | 44.2% |
|  |  | Bellow 9000 | 599 | 31.1% |
|  |  | Total | 1929 | 100.0% |
|  | Family structure | Nuclear | 1411 | 73.1% |
|  |  | Joint | 518 | 26.9% |
|  |  | Total | 1929 | 100.0% |
|  | Family residence | Independent | 474 | 24.6% |
|  |  | Matrilocal | 382 | 19.8% |
|  |  | Patrilocal | 1073 | 55.6% |
|  |  | Total | 1929 | 100.0% |
|  | Female Mobility Category | High | 570 | 29.5% |
|  |  | Moderate | 776 | 40.2% |
|  |  | Low | 583 | 30.2% |
|  |  | Total | 1929 | 100.0% |
|  | Female Empowerment | Good | 274 | 14.2% |
|  |  | Fair | 940 | 48.7% |
|  |  | Poor | 715 | 37.1% |
|  |  | Total | 1929 | 100.0% |

| **Continuous Variable Information** | | | | | | |
| --- | --- | --- | --- | --- | --- | --- |
|  | | N | Minimum | Maximum | Mean | Std. Deviation |
| Dependent Variable | Acceptance of Emotional Abuse | 1929 | .00 | 10.00 | 2.2628 | 2.23232 |

| **Goodness of Fit^a^** | | | |
| --- | --- | --- | --- |
|  | Value | df | Value/df |
| Deviance | 4053.835 | 1907 | 2.126 |
| Scaled Deviance | 4053.835 | 1907 |  |
| Pearson Chi-Square | 3698.572 | 1907 | 1.939 |
| Scaled Pearson Chi-Square | 3698.572 | 1907 |  |
| Log Likelihood^b^ | -3939.434 |  |  |
| Akaike's Information Criterion (AIC) | 7922.868 |  |  |
| Finite Sample Corrected AIC (AICC) | 7923.399 |  |  |
| Bayesian Information Criterion (BIC) | 8045.293 |  |  |
| Consistent AIC (CAIC) | 8067.293 |  |  |
| Dependent Variable: Acceptance of Emotional Abuse  Model: (Intercept), Ethnicity, Sex, Age.c, Edu.c, Occu, Familyinc.c, Structure, Residence, QCL_Moblty, EmpQCL_1^a^ | | | |
| a. Information criteria are in smaller-is-better form. | | | |
| b. The full log likelihood function is displayed and used in computing information criteria. | | | |

| **Omnibus Test^a^** | | |
| --- | --- | --- |
| Likelihood Ratio Chi-Square | df | Sig. |
| 799.390 | 21 | .000 |
| Dependent Variable: Acceptance of Emotional Abuse  Model: (Intercept), Ethnicity, Sex, Age.c, Edu.c, Occu, Familyinc.c, Structure, Residence, QCL_Moblty, EmpQCL_1^a^ | | |
| a. Compares the fitted model against the intercept-only model. | | |

| **Tests of Model Effects** | | | |
| --- | --- | --- | --- |
| Source | Type III | | |
|  | Wald Chi-Square | df | Sig. |
| (Intercept) | 213.792 | 1 | .000 |
| Ethnicity | 1.594 | 2 | .451 |
| Sex | 135.450 | 1 | .000 |
| Age.c | 6.812 | 3 | .078 |
| Edu.c | 18.240 | 3 | .000 |
| Occu | 5.485 | 3 | .140 |
| Familyinc.c | 1.694 | 2 | .429 |
| Structure | .229 | 1 | .632 |
| Residence | 3.598 | 2 | .165 |
| QCL_Moblty | 8.080 | 2 | .018 |
| EmpQCL_1 | 29.640 | 2 | .000 |
| Dependent Variable: Acceptance of Emotional Abuse  Model: (Intercept), Ethnicity, Sex, Age.c, Edu.c, Occu, Familyinc.c, Structure, Residence, QCL_Moblty, EmpQCL_1 | | | |

| **Parameter Estimates** | | | | | | | | | | |
| --- | --- | --- | --- | --- | --- | --- | --- | --- | --- | --- |
| Parameter | B | Std. Error | 95% Wald Confidence Interval | | Hypothesis Test | | | Exp(B) | 95% Wald Confidence Interval for Exp(B) | |
|  |  |  | Lower | Upper | Wald Chi-Square | df | Sig. |  | Lower | Upper |
| (Intercept) | 1.212 | .1683 | .883 | 1.542 | 51.894 | 1 | .000 | 3.362 | 2.417 | 4.676 |
| [Ethnicity=1.00] | .026 | .0768 | -.124 | .177 | .116 | 1 | .733 | 1.027 | .883 | 1.193 |
| [Ethnicity=2.00] | -.064 | .0678 | -.197 | .069 | .892 | 1 | .345 | .938 | .821 | 1.071 |
| [Ethnicity=3.00] | 0^a^ | . | . | . | . | . | . | 1 | . | . |
| [Sex=1.00] | -.819 | .0704 | -.957 | -.681 | 135.450 | 1 | .000 | .441 | .384 | .506 |
| [Sex=2.00] | 0^a^ | . | . | . | . | . | . | 1 | . | . |
| [Age.c=1.00] | -.129 | .1109 | -.347 | .088 | 1.360 | 1 | .244 | .879 | .707 | 1.092 |
| [Age.c=2.00] | -.042 | .0973 | -.233 | .149 | .184 | 1 | .668 | .959 | .793 | 1.161 |
| [Age.c=3.00] | .051 | .0926 | -.131 | .232 | .302 | 1 | .582 | 1.052 | .878 | 1.262 |
| [Age.c=4.00] | 0^a^ | . | . | . | . | . | . | 1 | . | . |
| [Edu.c=1.00] | -.066 | .1010 | -.264 | .132 | .430 | 1 | .512 | .936 | .768 | 1.141 |
| [Edu.c=2.00] | .151 | .0894 | -.024 | .326 | 2.867 | 1 | .090 | 1.163 | .976 | 1.386 |
| [Edu.c=3.00] | .191 | .0830 | .028 | .354 | 5.294 | 1 | .021 | 1.211 | 1.029 | 1.425 |
| [Edu.c=4.00] | 0^a^ | . | . | . | . | . | . | 1 | . | . |
| [Occu=1.00] | -.015 | .1123 | -.235 | .205 | .018 | 1 | .893 | .985 | .790 | 1.228 |
| [Occu=2.00] | .151 | .1157 | -.076 | .377 | 1.698 | 1 | .193 | 1.163 | .927 | 1.459 |
| [Occu=3.00] | .100 | .1106 | -.117 | .317 | .820 | 1 | .365 | 1.105 | .890 | 1.373 |
| [Occu=4.00] | 0^a^ | . | . | . | . | . | . | 1 | . | . |
| [Familyinc.c=1.00] | -.050 | .0590 | -.165 | .066 | .708 | 1 | .400 | .952 | .848 | 1.068 |
| [Familyinc.c=2.00] | .021 | .0492 | -.075 | .118 | .187 | 1 | .665 | 1.022 | .928 | 1.125 |
| [Familyinc.c=3.00] | 0^a^ | . | . | . | . | . | . | 1 | . | . |
| [Structure=1.00] | .026 | .0547 | -.081 | .133 | .229 | 1 | .632 | 1.027 | .922 | 1.143 |
| [Structure=2.00] | 0^a^ | . | . | . | . | . | . | 1 | . | . |
| [Residence=1.00] | .089 | .0516 | -.012 | .190 | 2.969 | 1 | .085 | 1.093 | .988 | 1.209 |
| [Residence=2.00] | -.017 | .0788 | -.171 | .138 | .045 | 1 | .832 | .983 | .843 | 1.148 |
| [Residence=3.00] | 0^a^ | . | . | . | . | . | . | 1 | . | . |
| [QCL_Moblty=1] | -.218 | .0781 | -.371 | -.065 | 7.762 | 1 | .005 | .804 | .690 | .938 |
| [QCL_Moblty=2] | -.068 | .0569 | -.179 | .044 | 1.417 | 1 | .234 | .935 | .836 | 1.045 |
| [QCL_Moblty=3] | 0^a^ | . | . | . | . | . | . | 1 | . | . |
| [EmpQCL_1=1] | -.326 | .0790 | -.481 | -.171 | 17.056 | 1 | .000 | .722 | .618 | .843 |
| [EmpQCL_1=2] | -.258 | .0514 | -.359 | -.158 | 25.259 | 1 | .000 | .772 | .698 | .854 |
| [EmpQCL_1=3] | 0^a^ | . | . | . | . | . | . | 1 | . | . |
| (Scale) | 1^b^ |  |  |  |  |  |  |  |  |  |
| Dependent Variable: Acceptance of Emotional Abuse  Model: (Intercept), Ethnicity, Sex, Age.c, Edu.c, Occu, Familyinc.c, Structure, Residence, QCL_Moblty, EmpQCL_1 | | | | | | | | | | |
| a. Set to zero because this parameter is redundant. | | | | | | | | | | |
| b. Fixed at the displayed value. | | | | | | | | | | |

**Estimated Marginal Means 1: Grand Mean**

| **Estimates** | | | |
| --- | --- | --- | --- |
| Mean | Std. Error | 95% Wald Confidence Interval | |
|  |  | Lower | Upper |
| 1.8701 | .08007 | 1.7196 | 2.0338 |

**Estimated Marginal Means 2: Ethnicity**

| **Estimates** | | | | |
| --- | --- | --- | --- | --- |
| Ethnicity | Mean | Std. Error | 95% Wald Confidence Interval | |
|  |  |  | Lower | Upper |
| Garo | 1.9441 | .10437 | 1.7499 | 2.1598 |
| Santal | 1.7764 | .11516 | 1.5645 | 2.0171 |
| Bengali | 1.8938 | .11817 | 1.6758 | 2.1402 |

* Generalized Linear Models.

GENLIN AcAbs BY Ethnicity Sex Age.c Edu.c Occu Familyinc.c Structure Residence QCL_Moblty EmpQCL_1

(ORDER=ASCENDING)

/MODEL Ethnicity Sex Age.c Edu.c Occu Familyinc.c Structure Residence QCL_Moblty EmpQCL_1

INTERCEPT=YES

DISTRIBUTION=POISSON LINK=LOG

/CRITERIA METHOD=FISHER(1) SCALE=1 COVB=ROBUST MAXITERATIONS=100 MAXSTEPHALVING=5

PCONVERGE=1E-006(ABSOLUTE) SINGULAR=1E-012 ANALYSISTYPE=3(WALD) CILEVEL=95 CITYPE=WALD

LIKELIHOOD=FULL

/EMMEANS SCALE=ORIGINAL

/EMMEANS TABLES=Ethnicity SCALE=ORIGINAL

/MISSING CLASSMISSING=EXCLUDE

/PRINT CPS DESCRIPTIVES MODELINFO FIT SUMMARY SOLUTION (EXPONENTIATED).

**Generalized Linear Models**

| **Notes** | | |
| --- | --- | --- |
| Output Created | | 21-MAY-2020 18:11:41 |
| Comments | |  |
| Input | Data | F:\Lineas\Paper 2\Baseline Data May 2019_1 (04-7-19 Final).sav |
|  | Active Dataset | DataSet1 |
|  | Filter | <none> |
|  | Weight | <none> |
|  | Split File | <none> |
|  | N of Rows in Working Data File | 1929 |
| Missing Value Handling | Definition of Missing | User-defined missing values for factor, subject and within-subject variables are treated as missing. |
|  | Cases Used | Statistics are based on cases with valid data for all variables in the model. |
| Weight Handling | | not applicable |
| Syntax | | GENLIN AcAbs BY Ethnicity Sex Age.c Edu.c Occu Familyinc.c Structure Residence QCL_Moblty EmpQCL_1  (ORDER=ASCENDING)  /MODEL Ethnicity Sex Age.c Edu.c Occu Familyinc.c Structure Residence QCL_Moblty EmpQCL_1  INTERCEPT=YES  DISTRIBUTION=POISSON LINK=LOG  /CRITERIA METHOD=FISHER(1) SCALE=1 COVB=ROBUST MAXITERATIONS=100 MAXSTEPHALVING=5  PCONVERGE=1E-006(ABSOLUTE) SINGULAR=1E-012 ANALYSISTYPE=3(WALD) CILEVEL=95 CITYPE=WALD  LIKELIHOOD=FULL  /EMMEANS SCALE=ORIGINAL  /EMMEANS TABLES=Ethnicity SCALE=ORIGINAL  /MISSING CLASSMISSING=EXCLUDE  /PRINT CPS DESCRIPTIVES MODELINFO FIT SUMMARY SOLUTION (EXPONENTIATED). |
| Resources | Processor Time | 00:00:00.25 |
|  | Elapsed Time | 00:00:00.28 |

| **Model Information** | |
| --- | --- |
| Dependent Variable | Acceptance of Any Abuse |
| Probability Distribution | Poisson |
| Link Function | Log |

| **Case Processing Summary** | | |
| --- | --- | --- |
|  | N | Percent |
| Included | 1929 | 100.0% |
| Excluded | 0 | 0.0% |
| Total | 1929 | 100.0% |

| **Categorical Variable Information** | | | | |
| --- | --- | --- | --- | --- |
|  | | | N | Percent |
| Factor | Ethnicity | Garo | 640 | 33.2% |
|  |  | Santal | 640 | 33.2% |
|  |  | Bengali | 649 | 33.6% |
|  |  | Total | 1929 | 100.0% |
|  | Sex | Male | 960 | 49.8% |
|  |  | Female | 969 | 50.2% |
|  |  | Total | 1929 | 100.0% |
|  | Age in years | 16-25 | 301 | 15.6% |
|  |  | 26-35 | 746 | 38.7% |
|  |  | 36-45 | 711 | 36.9% |
|  |  | 46-60 | 171 | 8.9% |
|  |  | Total | 1929 | 100.0% |
|  | Schooling | Upper | 385 | 20.0% |
|  |  | Secondary | 575 | 29.8% |
|  |  | Primary | 820 | 42.5% |
|  |  | None | 149 | 7.7% |
|  |  | Total | 1929 | 100.0% |
|  | Occupation | Unemployed | 636 | 33.0% |
|  |  | Agric farming | 329 | 17.1% |
|  |  | Day laborers | 797 | 41.3% |
|  |  | Job and others | 167 | 8.7% |
|  |  | Total | 1929 | 100.0% |
|  | Family Income | 15000/above | 478 | 24.8% |
|  |  | 9000-14999 | 852 | 44.2% |
|  |  | Bellow 9000 | 599 | 31.1% |
|  |  | Total | 1929 | 100.0% |
|  | Family structure | Nuclear | 1411 | 73.1% |
|  |  | Joint | 518 | 26.9% |
|  |  | Total | 1929 | 100.0% |
|  | Family residence | Independent | 474 | 24.6% |
|  |  | Matrilocal | 382 | 19.8% |
|  |  | Patrilocal | 1073 | 55.6% |
|  |  | Total | 1929 | 100.0% |
|  | Female Mobility Category | High | 570 | 29.5% |
|  |  | Moderate | 776 | 40.2% |
|  |  | Low | 583 | 30.2% |
|  |  | Total | 1929 | 100.0% |
|  | Female Empowerment | Good | 274 | 14.2% |
|  |  | Fair | 940 | 48.7% |
|  |  | Poor | 715 | 37.1% |
|  |  | Total | 1929 | 100.0% |

| **Continuous Variable Information** | | | | | | |
| --- | --- | --- | --- | --- | --- | --- |
|  | | N | Minimum | Maximum | Mean | Std. Deviation |
| Dependent Variable | Acceptance of Any Abuse | 1929 | .00 | 10.00 | 3.0187 | 2.83807 |

| **Goodness of Fit^a^** | | | |
| --- | --- | --- | --- |
|  | Value | df | Value/df |
| Deviance | 4518.764 | 1907 | 2.370 |
| Scaled Deviance | 4518.764 | 1907 |  |
| Pearson Chi-Square | 4217.466 | 1907 | 2.212 |
| Scaled Pearson Chi-Square | 4217.466 | 1907 |  |
| Log Likelihood^b^ | -4425.505 |  |  |
| Akaike's Information Criterion (AIC) | 8895.010 |  |  |
| Finite Sample Corrected AIC (AICC) | 8895.541 |  |  |
| Bayesian Information Criterion (BIC) | 9017.435 |  |  |
| Consistent AIC (CAIC) | 9039.435 |  |  |
| Dependent Variable: Acceptance of Any Abuse  Model: (Intercept), Ethnicity, Sex, Age.c, Edu.c, Occu, Familyinc.c, Structure, Residence, QCL_Moblty, EmpQCL_1^a^ | | | |
| a. Information criteria are in smaller-is-better form. | | | |
| b. The full log likelihood function is displayed and used in computing information criteria. | | | |

| **Omnibus Test^a^** | | |
| --- | --- | --- |
| Likelihood Ratio Chi-Square | df | Sig. |
| 1395.001 | 21 | .000 |
| Dependent Variable: Acceptance of Any Abuse  Model: (Intercept), Ethnicity, Sex, Age.c, Edu.c, Occu, Familyinc.c, Structure, Residence, QCL_Moblty, EmpQCL_1^a^ | | |
| a. Compares the fitted model against the intercept-only model. | | |

| **Tests of Model Effects** | | | |
| --- | --- | --- | --- |
| Source | Type III | | |
|  | Wald Chi-Square | df | Sig. |
| (Intercept) | 574.532 | 1 | .000 |
| Ethnicity | 9.287 | 2 | .010 |
| Sex | 221.067 | 1 | .000 |
| Age.c | 9.649 | 3 | .022 |
| Edu.c | 21.484 | 3 | .000 |
| Occu | 9.234 | 3 | .026 |
| Familyinc.c | 2.920 | 2 | .232 |
| Structure | .001 | 1 | .972 |
| Residence | 3.408 | 2 | .182 |
| QCL_Moblty | 11.009 | 2 | .004 |
| EmpQCL_1 | 22.208 | 2 | .000 |
| Dependent Variable: Acceptance of Any Abuse  Model: (Intercept), Ethnicity, Sex, Age.c, Edu.c, Occu, Familyinc.c, Structure, Residence, QCL_Moblty, EmpQCL_1 | | | |

| **Parameter Estimates** | | | | | | | | | | |
| --- | --- | --- | --- | --- | --- | --- | --- | --- | --- | --- |
| Parameter | B | Std. Error | 95% Wald Confidence Interval | | Hypothesis Test | | | Exp(B) | 95% Wald Confidence Interval for Exp(B) | |
|  |  |  | Lower | Upper | Wald Chi-Square | df | Sig. |  | Lower | Upper |
| (Intercept) | 1.913 | .1573 | 1.604 | 2.221 | 147.826 | 1 | .000 | 6.772 | 4.975 | 9.218 |
| [Ethnicity=1.00] | -.179 | .0709 | -.318 | -.040 | 6.376 | 1 | .012 | .836 | .728 | .961 |
| [Ethnicity=2.00] | -.156 | .0621 | -.278 | -.034 | 6.295 | 1 | .012 | .856 | .758 | .966 |
| [Ethnicity=3.00] | 0^a^ | . | . | . | . | . | . | 1 | . | . |
| [Sex=1.00] | -1.005 | .0676 | -1.137 | -.872 | 221.067 | 1 | .000 | .366 | .321 | .418 |
| [Sex=2.00] | 0^a^ | . | . | . | . | . | . | 1 | . | . |
| [Age.c=1.00] | -.245 | .1043 | -.449 | -.040 | 5.511 | 1 | .019 | .783 | .638 | .960 |
| [Age.c=2.00] | -.189 | .0943 | -.374 | -.004 | 4.011 | 1 | .045 | .828 | .688 | .996 |
| [Age.c=3.00] | -.081 | .0892 | -.256 | .094 | .820 | 1 | .365 | .922 | .774 | 1.099 |
| [Age.c=4.00] | 0^a^ | . | . | . | . | . | . | 1 | . | . |
| [Edu.c=1.00] | -.253 | .0872 | -.424 | -.082 | 8.442 | 1 | .004 | .776 | .654 | .921 |
| [Edu.c=2.00] | .005 | .0737 | -.139 | .150 | .005 | 1 | .942 | 1.005 | .870 | 1.162 |
| [Edu.c=3.00] | .035 | .0685 | -.100 | .169 | .256 | 1 | .613 | 1.035 | .905 | 1.184 |
| [Edu.c=4.00] | 0^a^ | . | . | . | . | . | . | 1 | . | . |
| [Occu=1.00] | .001 | .1067 | -.208 | .210 | .000 | 1 | .994 | 1.001 | .812 | 1.234 |
| [Occu=2.00] | .190 | .1111 | -.027 | .408 | 2.932 | 1 | .087 | 1.209 | .973 | 1.504 |
| [Occu=3.00] | .138 | .1068 | -.072 | .347 | 1.661 | 1 | .198 | 1.148 | .931 | 1.415 |
| [Occu=4.00] | 0^a^ | . | . | . | . | . | . | 1 | . | . |
| [Familyinc.c=1.00] | -.080 | .0542 | -.186 | .026 | 2.165 | 1 | .141 | .923 | .830 | 1.027 |
| [Familyinc.c=2.00] | .001 | .0441 | -.086 | .087 | .000 | 1 | .983 | 1.001 | .918 | 1.091 |
| [Familyinc.c=3.00] | 0^a^ | . | . | . | . | . | . | 1 | . | . |
| [Structure=1.00] | .002 | .0483 | -.093 | .096 | .001 | 1 | .972 | 1.002 | .911 | 1.101 |
| [Structure=2.00] | 0^a^ | . | . | . | . | . | . | 1 | . | . |
| [Residence=1.00] | .083 | .0452 | -.006 | .172 | 3.365 | 1 | .067 | 1.087 | .994 | 1.187 |
| [Residence=2.00] | .029 | .0750 | -.118 | .176 | .153 | 1 | .696 | 1.030 | .889 | 1.193 |
| [Residence=3.00] | 0^a^ | . | . | . | . | . | . | 1 | . | . |
| [QCL_Moblty=1] | -.226 | .0698 | -.363 | -.089 | 10.495 | 1 | .001 | .798 | .696 | .915 |
| [QCL_Moblty=2] | -.117 | .0495 | -.214 | -.020 | 5.568 | 1 | .018 | .890 | .808 | .980 |
| [QCL_Moblty=3] | 0^a^ | . | . | . | . | . | . | 1 | . | . |
| [EmpQCL_1=1] | -.234 | .0714 | -.374 | -.094 | 10.741 | 1 | .001 | .791 | .688 | .910 |
| [EmpQCL_1=2] | -.206 | .0461 | -.296 | -.115 | 19.930 | 1 | .000 | .814 | .744 | .891 |
| [EmpQCL_1=3] | 0^a^ | . | . | . | . | . | . | 1 | . | . |
| (Scale) | 1^b^ |  |  |  |  |  |  |  |  |  |
| Dependent Variable: Acceptance of Any Abuse  Model: (Intercept), Ethnicity, Sex, Age.c, Edu.c, Occu, Familyinc.c, Structure, Residence, QCL_Moblty, EmpQCL_1 | | | | | | | | | | |
| a. Set to zero because this parameter is redundant. | | | | | | | | | | |
| b. Fixed at the displayed value. | | | | | | | | | | |

**Estimated Marginal Means 1: Grand Mean**

| **Estimates** | | | |
| --- | --- | --- | --- |
| Mean | Std. Error | 95% Wald Confidence Interval | |
|  |  | Lower | Upper |
| 2.5860 | .10251 | 2.3927 | 2.7950 |

**Estimated Marginal Means 2: Ethnicity**

| **Estimates** | | | | |
| --- | --- | --- | --- | --- |
| Ethnicity | Mean | Std. Error | 95% Wald Confidence Interval | |
|  |  |  | Lower | Upper |
| Garo | 2.4177 | .12070 | 2.1923 | 2.6662 |
| Santal | 2.4740 | .15512 | 2.1879 | 2.7975 |
| Bengali | 2.8914 | .16206 | 2.5906 | 3.2271 |
